# Zika virus non-structural protein NS4A restricts eye growth in *Drosophila* through regulation of JAK/STAT signaling

**DOI:** 10.1242/dmm.040816

**Published:** 2020-04-30

**Authors:** Sneh Harsh, Yulong Fu, Eric Kenney, Zhe Han, Ioannis Eleftherianos

**Affiliations:** 1 Department of Biological Sciences, The George Washington University, Washington, DC 20052, USA; 2 NYU Langone Health, Alexandria Center for Life Science, New York, NY 10016, USA; 3 Center for Genetic Medicine Research, Children's National Health System. Department of Genomics and Precision Medicine, The George Washington University School of Medicine and Health Sciences, Washington, DC 20010, USA

**Keywords:** *Drosophila*, Eye development, JAK/STAT signaling, Host-pathogen interaction, Zika virus

## Abstract

To gain a comprehensive view of the changes in host gene expression underlying Zika virus (ZIKV) pathogenesis, we performed whole-genome RNA sequencing (RNA-seq) of ZIKV-infected *Drosophila* adult flies. RNA-seq analysis revealed that ZIKV infection alters several and diverse biological processes, including stress, locomotion, lipid metabolism, imaginal disc morphogenesis and regulation of JAK/STAT signaling. To explore the interaction between ZIKV infection and JAK/STAT signaling regulation, we generated genetic constructs overexpressing ZIKV-specific non-structural proteins NS2A, NS2B, NS4A and NS4B. We found that ectopic expression of non-structural proteins in the developing *Drosophila* eye significantly restricts growth of the larval and adult eye and correlates with considerable repression of the *in vivo* JAK/STAT reporter, *10XStat92E-GFP*. At the cellular level, eye growth defects are associated with reduced rate of proliferation without affecting the overall rate of apoptosis. In addition, ZIKV NS4A genetically interacts with the JAK/STAT signaling components; co-expression of *NS4A* along with the dominant-negative form of *domeless* or *StatRNAi* results in aggravated reduction in eye size, while co-expression of *NS4A* in *HopTuml* (also known as *hop^Tum^*) mutant background partially rescues the *hop*-induced eye overgrowth phenotype. The function of ZIKV NS4A in regulating growth is maintained in the wing, where ZIKV *NS4A* overexpression in the pouch domain results in reduced growth linked with diminished expression of Notch targets, Wingless (Wg) and Cut, and the Notch reporter, *NRE-GFP*. Thus, our study provides evidence that ZIKV infection in *Drosophila* results in restricted growth of the developing eye and wing, wherein eye phenotype is induced through regulation of JAK/STAT signaling, whereas restricted wing growth is induced through regulation of Notch signaling. The interaction of ZIKV non-structural proteins with the conserved host signaling pathways further advance our understanding of ZIKV-induced pathogenesis.

This article has an associated First Person interview with the first author of the paper.

## Abstract (precis)

**Summary:** Our study provides evidence that Zika virus infection in *Drosophila* results in restricted growth of the developing eye and wing through regulation of JAK/STAT and Notch signaling, respectively.

## INTRODUCTION

Zika virus (ZIKV) is an emerging pathogen of substantial public health concern, and belongs to the flavivirus family that also includes dengue, West Nile and yellow fever viruses. Although most infections are asymptomatic or associated with flu-like symptoms (Simpson, 1964), during the recent epidemic, ZIKV has been associated with multi-organ failure resulting in congenital abnormalities in fetuses of pregnant women and neurological complications (Guillain-Barre syndrome) characterized by progressive muscle weakness (Dos Santos et al., 2016; do Rosario et al., 2016). ZIKV can also infect the eye, resulting in conjunctivitis in up to 15% of the patients (Sun et al., 2016; Miner et al., 2016; Furtado et al., 2016). Although ZIKV is primarily transmitted by mosquitos, perinatal and congenital infections, infection through blood transfusion as well as sexual transmission have also been reported (Musso et al., 2014; Mead et al., 2018; Foy et al., 2011). In particular, it is the correlation of ZIKV-associated outbreak with microcephaly that makes ZIKA infection even more serious (Li et al., 2016a). Until now, no drug or vaccine is available to prevent or treat ZIKV infection (Fauci and Morens, 2016).

ZIKV is an obligate intracellular pathogen; therefore, understanding the interaction dynamics between ZIKV and the host and the resulting host pathology is valuable for developing anti-ZIKV therapeutic strategies (Shirasu-Hiza and Schneider, 2007). In particular, the symptoms or physiological consequences of infection prior to disease development reflect the physical state of the organism and contribute to the successful completion of the virus life cycle. These host-virus interactions and the resulting host response are equally important in defining the pathological outcome of an infection. Although efforts have been made to understand the intricacies of ZIKV replication, there have been very few pieces of evidence showing the ZIKV effects on host physiology (Wang et al., 2018; Liang et al., 2016).

Investigating the function of viral components is an elegant strategy to further our understanding of the molecular basis of viral diseases. Structurally, ZIKV is similar to the other flaviviruses and possesses a 25- to 30-nm nucleocapsid surrounded by a host membrane-derived lipid bilayer. ZIKV contains a positive-sense single-stranded RNA of ∼10 kb. Similar to other flaviviruses, host protease-mediated viral processing results in three structural (capsid, pre-membrane, envelope) and seven non-structural (NS) proteins (NS1, NS2A, NS2B, NS3, NS4A, NS4B, NS5) (Chambers et al., 1990; Wang et al., 2017). In other flaviviruses, the NS proteins are required for viral replication and immune evasion, specifically through interference with RIG-I like receptor (RLR) signaling and type I interferon response (Ngono and Shresta, 2018; Chen et al., 2017). NS2 from dengue and Kunjin virus inhibits interferon-mediated response (Liu et al., 2004; Dalrymple et al., 2015). NS4A and NS4B from flaviviruses are able to inhibit Janus kinase/Signal transducer and activators of transcription (JAK/STAT) and RLR signaling through multiple mechanisms (Ding et al., 2013; Munoz-Jordan et al., 2003). However, to date, there is little information on the functional significance of ZIKV NS proteins and their role in ZIKV-induced pathogenesis. In an earlier study, overexpression of ZIKV NS proteins NS4A and NS4B in the fetal neuronal stem cells (fNSCs) reduced neurosphere formation and inhibited differentiation (Liang et al., 2016). ZIKV NS4A- and NS4B-mediated effect on neurogenesis was further linked to increased autophagy mediated by Akt-mTOR signaling (Liang et al., 2016). This result was specific to ZIKV, as NS4A and NS4B from the closely related dengue virus failed to show a similar effect (Liang et al., 2016).

The common fruit fly, *Drosophila melanogaster*, with a vast number of genetic and genomic tools available and highly conserved developmental signaling pathways, is widely recognized as an excellent model for studying host-pathogen interactions and human disease. The latter is demonstrated by the fact that 70% of *Drosophila* genes have human homologs and 75% of human disease-associated genes have homologs in the fly (Bernards and Hariharan, 2001; Edgar and Lehner, 1996; Pandey and Nichols, 2011). Evidence indicates that the fly is also a suitable model for dissecting pathologies related to human pathogenic viruses (Xu and Cherry, 2014; Hughes et al., 2012). In addition, *Drosophila* can be efficiently used to underscore the *in vivo* function of viral genes, which are further validated in mammalian models (Hughes et al., 2012). Using the Gal4/UAS system, viral transgenes can be expressed in a spatial and temporal manner followed by the analysis of the resulting phenotype. For instance, overexpression of SARS-CoV 3a and SARS-CoV membrane proteins induces apoptosis in the developing eye through the mitochondrial pathway via Cytochrome c and suppressing survival signaling pathways, respectively (Wong et al., 2005; Chan et al., 2007). Similarly, overexpression of human immunodeficiency virus (HIV) *nef* gene in the wing results in increased apoptosis without affecting the rate of proliferation (Lee et al., 2005). Overexpression of human cytomegalovirus immediate-early genes in *Drosophila* embryos results in abnormal embryonic development associated with disrupted adherens junctions (Steinberg et al., 2008). Recently, there have also been efforts to dissect host-ZIKV interactions using *Drosophila* as the model organism (Harsh et al., 2018; Link et al., 2019; Liu et al., 2018). Findings from these studies indicate that *Drosophila* can be a reliable model to analyze ZIKV tropism. ZIKV was shown to replicate in the fat body, midgut, crop and brain of the infected adult fly, and result in perturbation in lipid metabolism, intestinal homeostasis and autophagy (Liu et al., 2018; Harsh et al., 2018).

Here, we have challenged *Drosophila* adult flies with the MR766 strain of ZIKV and used RNA sequencing (RNA-seq) to gain a comprehensive understanding of the differentially regulated genes during infection. We report that ZIKV triggers a large number of biological processes, ranging from misregulation of developmental pathways to perturbed muscle development. Among the developmental signaling pathways, we have shown that ZIKV infection induces negative regulation of JAK/STAT signaling. Eye-specific expression of ZIKV transgenes NS2A, NS2B, NS4A and NS4B results in striking reduction in the size of the developing eye. We also show that the reduced eye size upon ZIKV NS4A protein expression, in particular, correlates with the reduced level of the JAK/STAT reporter, *10XStat92E-GFP*. At the cellular level, the expression of ZIKV transgenes results in reduced rate of proliferation in the JAK/STAT-regulated anterior compartment of eye imaginal epithelia without affecting the level of apoptosis. We further demonstrate that ZIKV transgene expression has no effect on the differentiation of the photoreceptors of the eye, and that ZIKV NS4A interacts with the JAK/STAT signaling components. Co-expression of NS4A and the dominant-negative form of *domeless* or *StatRNAi* results in aggravated reduction in eye size, while co-expression of NS4A with activated Hop kinase partially rescues the eye enlargement. Finally, the ZIKV NS4A-mediated regulation of growth is also maintained in the wing, where NS4A overexpression restricts the size of the wing pouch. This effect is linked with reduced activity of Notch signaling, which is instrumental for several cell developmental processes, including proliferation and wing development. Altogether, our findings provide the first evidence linking ZIKV-induced pathogenesis and eye/wing development, and reveal a functional link between ZIKV and regulation of JAK/STAT signaling.

## RESULTS

### ZIKV infection induces distinct transcriptomic profiles in *Drosophila*

**Fig. 1.**
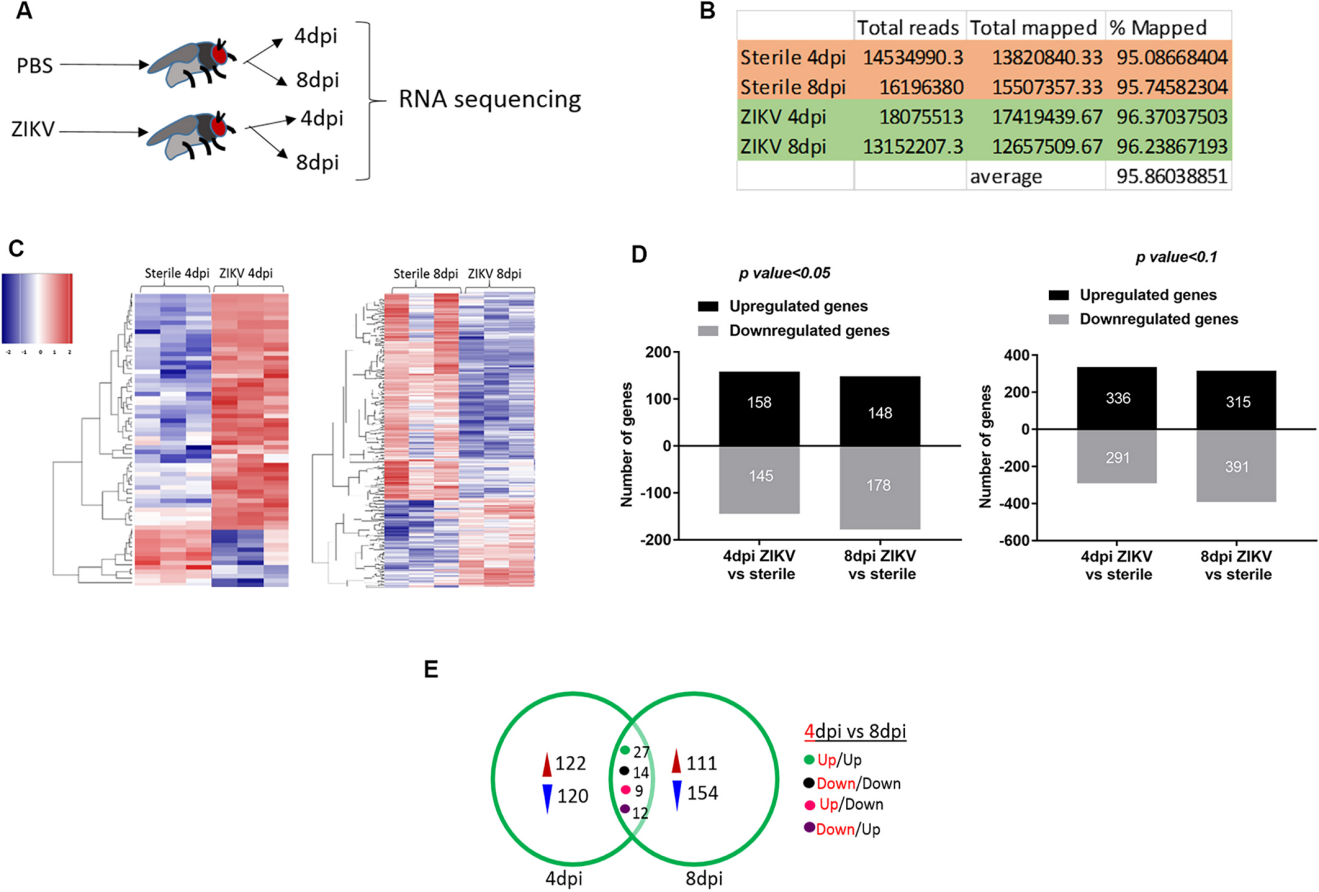
**Transcriptome analysis of ZIKV-infected flies through RNA-seq.** (A) Overview of the experimental workflow. 5- to 6-day-old wild-type female flies (*w^1118^* strain) were injected with the MR766 strain of ZIKV. PBS-injected flies served as negative controls. Total RNA was extracted at 4 and 8 days post-injection (dpi) for RNA-seq. (B) Transcriptome summary (number of reads and percentage mapped to *D. melanogaster* genome) from wild-type flies injected with PBS and ZIKV at 4 and 8 dpi. (C) Heat map showing the differentially expressed genes (DEGs) in PBS- and ZIKV-injected flies (in triplicate) at 4 and 8 dpi. (D) DEGs (downregulated/upregulated) in wild-type flies injected with ZIKV at 4 and 8 dpi when adjusted *P*-value is set at 0.05 and 0.1, respectively. (E) Venn diagrams showing the number of *Drosophila* genes that are differentially expressed (upregulated or downregulated) in wild-type flies injected with ZIKV at 4 and 8 dpi. Expression patterns are indicated (Up/Up, gene upregulation at both 4 and 8 dpi; Down/Down, gene downregulation at both timepoints; Up/Down, gene upregulation at 4 dpi and downregulation at 8 dpi; Down/Up, gene downregulation at 4 dpi and upregulation at 8 dpi). Significance was tested using a hypergeometric test: *P*-values for Up/Up, Down/Down, Up/Down and Down/Up trending genes were 2.065×10^–33^, 1.361×10^–11^, 3.050×10^–9^ and 5.485×10^–11^, respectively.

We generated complete transcriptomes from *Drosophila* wild-type female adult flies infected with the MR766 strain of ZIKV (Harsh et al., 2018; Liu et al., 2018) or injected with PBS (negative/sterile control) (Harsh et al., 2018). We examined gene transcript levels at two timepoints, 4 and 8 days post-injection (dpi) (Fig. 1A). The timepoints were chosen based on the ZIKV load, where 4 dpi corresponds to the early stage of infection, while 8 dpi corresponds to the stage when the infection reaches peak titers (Harsh et al., 2018). The numbers of sequence reads mapped to 96.37% of the *D. melanogaster* genome (Fig. 1B). Differences in gene expression levels across the two treatments, timepoints and technical replicates are illustrated in the form of a heat map (Fig. 1C). There was a substantial difference in the number of differentially expressed genes (DEGs) when the adjusted *P*-value was kept at 0.1 versus 0.05 (Fig. 1D). In particular, the number of upregulated genes in ZIKV-injected flies compared to PBS-injected flies increased from 158 to 336 at 4 dpi, and from 148 to 315 at 8 dpi (Fig. 1D). To determine the number of genes that were transcriptionally regulated upon infection with ZIKV at 4 and 8 dpi, we performed pairwise multiple comparison analyses with Limma (Liu et al., 2015; Ritchie et al., 2015). We found that the number of differentially regulated genes varied between 4 and 8 dpi (Fig. 1E). The number of upregulated genes at both 4 and 8 dpi was higher than the number of downregulated genes upon ZIKV infection (Fig. 1E). These results suggest that a large set of genes is differentially regulated in *Drosophila* adult flies during the early and late stages of infection with ZIKV.

### ZIKV infection leads to the enrichment of genes associated with diverse biological processes in *Drosophila*

**Fig. 2.**
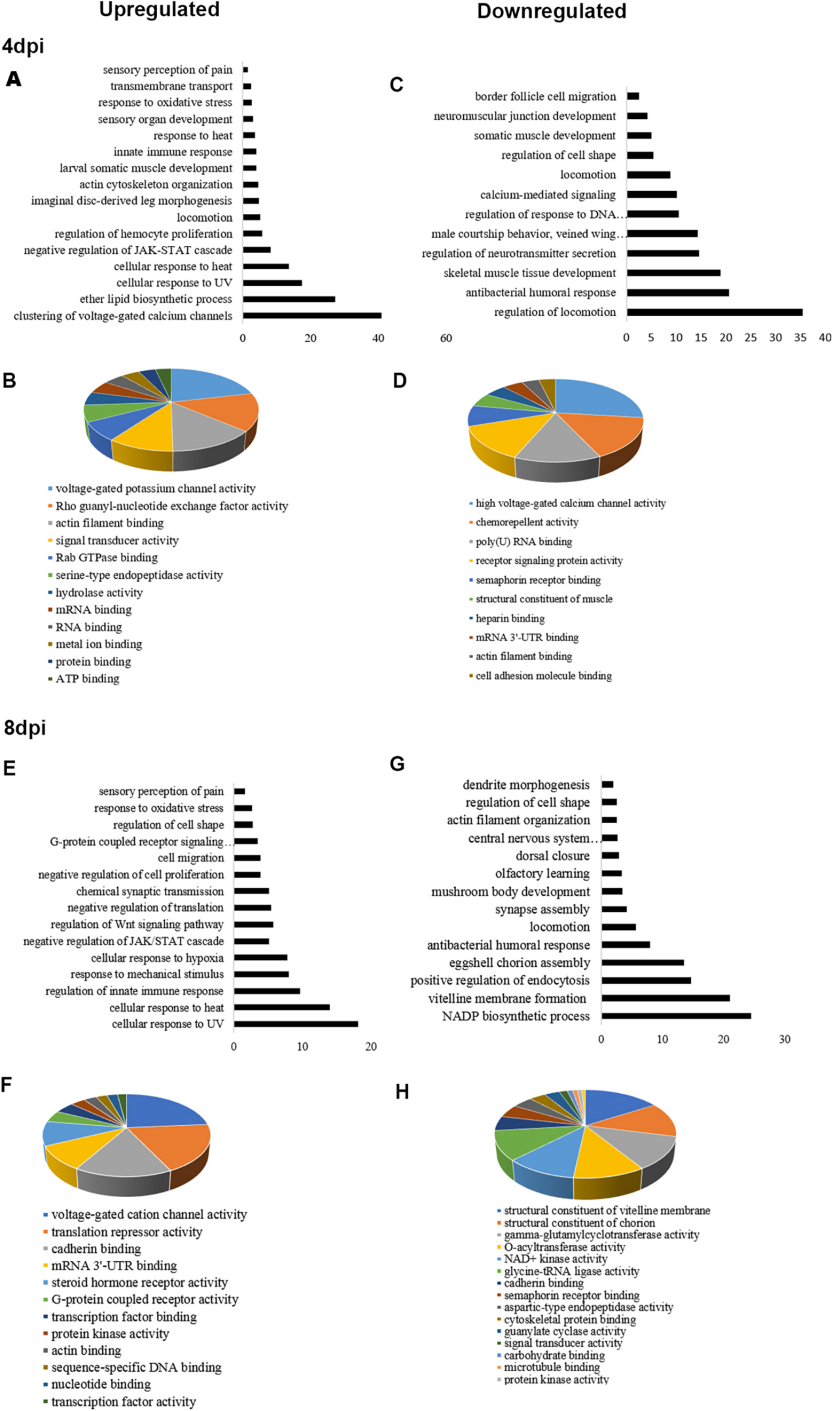
**Infection of adult flies with ZIKV induces diverse biological processes and molecular functions.** (A,E) Representative enrichment of upregulated (log>1.2 fold) biological processes using Database for Annotation, Visualization and Integrated Discovery (DAVID) classification database at 4 and 8 dpi. (C,G) Representative enrichment of downregulated (log<−1.2 fold) biological processes using DAVID at 4 and 8 dpi. (B,D,F,H) GO-based molecular functions regulated by the DEGs at 4 dpi (B,D) and 8 dpi (F,H).

To identify the molecular functions and biological activities regulated by ZIKV in *Drosophila*, we performed Database for Annotation, Visualization and Integrated Discovery (DAVID)-based analyses (Huang da et al., 2009a,b) (Fig. 2). At both 4 and 8 dpi, there was considerable enrichment in the number of genes controlling biological processes and molecular functions (Fig. 2). In order to retrieve a significant number of DEGs, we set a log2 fold change of 1.2 as an arbitrary cutoff threshold and adjusted *P*-value at 0.1 (Fig. 1D). This cutoff led to identification of 627 DEGs at 4 dpi, of which 336 were upregulated and 291 were downregulated (Tables S1 and S2). At 8 dpi, 706 genes were differentially expressed, of which 315 genes were upregulated and 391 genes were downregulated (Tables S3 and S4). Among the DEGs regulating immunity, *Diedel* and genes of the Turandot family of proteins were significantly enriched at both 4 and 8 dpi (Fig. 2A,E; Tables S1 and S3). Unlike for *Drosophila* C virus (DCV) infection (Merkling et al., 2015), we did not encounter enrichment of heat shock proteins at any stage of infection. However, gene ontology (GO) terms such as ‘response to heat, UV and hypoxia’ were significantly enriched at both 4 and 8 dpi (Fig. 2A,E). Another set of genes enriched at both 4 and 8 dpi included genes regulating muscle development (Fig. 2A,E). Interestingly, ZIKV infection also resulted in enrichment of genes regulating developmental pathways, and, in particular, JAK/STAT and Wnt signaling pathways (Fig. 2A,E). Genes that were downregulated at both 4 and 8 dpi were enriched for GO terms such as central nervous system development or synapse assembly (Fig. 2C,G). Genes regulating antibacterial humoral immune response and egg chorion assembly were specifically downregulated at 8 dpi (Fig. 2G). In the context of molecular function, the genes enriched at 4 and 8 dpi were mostly associated with signal transducer activity, actin binding, cadherin binding and structural constituent of chorion (Fig. 2B,D,F,H). Together, these data suggest that ZIKV infection triggers some of the conserved microbe-specific responses, including immune and stress response, and also activates some specific processes related to the regulation of developmental pathways, such as JAK/STAT signaling and vitelline assembly.

### qRT-PCR-based transcriptome analysis reveals upregulation of negative regulators of JAK/STAT signaling upon ZIKV infection

**Fig. 3.**
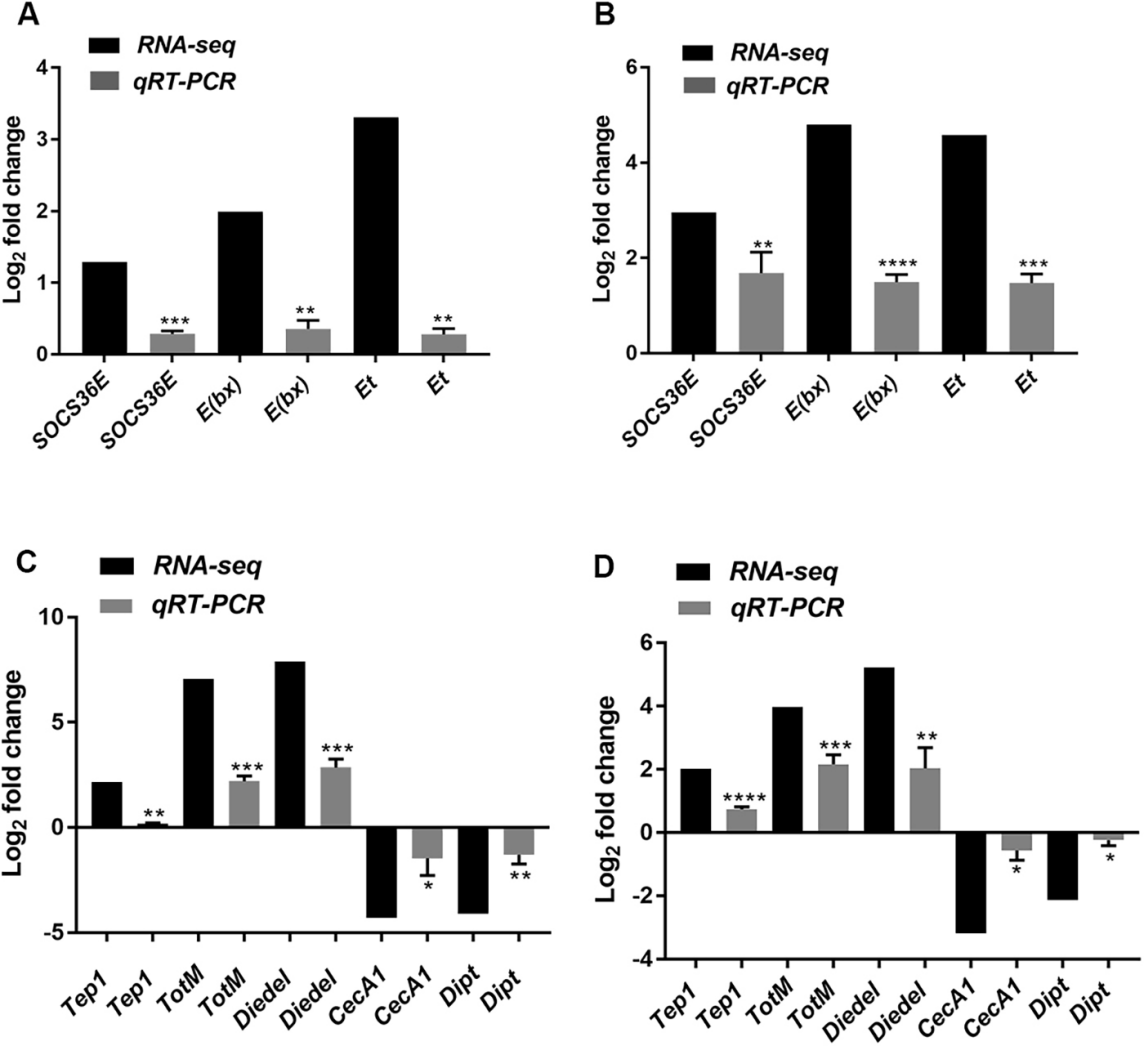
**Validation of the enriched negative regulators of JAK/STAT pathway by qRT-PCR.** (A,B) Log2 fold change (RNA-seq) and mRNA levels (qRT-PCR) of *Socs36E*, *E(bx)* and *Et* in wild-type adult flies infected with ZIKV at 4 and 8 dpi. (C,D) Log2 fold change (RNA-seq) and mRNA levels (qRT-PCR) of *Tep1*, *TotM*, *Diedel*, *CecA1* and *Dipt* in wild-type adult flies infected with ZIKV at 4 and 8 dpi. All data were normalized to the housekeeping gene *RpL32* and are shown relative to wild-type flies injected with PBS (sterile control). Three independent experiments were carried out with ten flies per sample in triplicate (‘*P<0.05, **P<0.01, ***P<0.001, ****P<0.0001). Bars represent the mean±s.d. Statistical analysis was performed using unpaired two-tailed Student's *t*-test.

To validate our RNA-seq results (Fig. 2), we performed quantitative real-time PCR (qRT-PCR) on *Drosophila* flies infected with ZIKV at 4 and 8 dpi (Fig. 3). Interestingly, we found that one of the prominent ZIKV infection-induced processes relates to the negative regulation of JAK/STAT signaling. First identified as a key regulator of interferon and cytokine signaling in mammals, JAK/STAT signaling regulates pleiotropic effects including growth and differentiation (Schindler et al., 1992; Shuai et al., 1994, 1992; Velazquez et al., 1992; Watling et al., 1993). JAK/STAT signaling is also known to regulate cellular proliferation, immune responses and maintenance, and proliferation of stem cells in the gonads (reviewed in Arbouzova and Zeidler, 2006). Given the range of biological roles played by JAK/STAT signaling, it is not surprising that this pathway is controlled through multiple regulatory mechanisms. The wide range of phenotypes associated with the expression of negative regulators of JAK/STAT signaling prompted us to validate the RNA-seq results.

We noticed that the established negative regulators of JAK/STAT signaling did not show significant upregulation (in terms of the adjusted *P*-value). Also, comparing the individual fold change in all three biological replicates revealed that, out of the three replicates, one replicate deviated from the other two replicates, resulting in the higher *P*-value and hence non-significant upregulation (Table S5). However, consistent with several pieces of evidence showing the interaction of flaviviruses with the regulators of JAK/STAT signaling (Laurent-Rolle et al., 2010; Kumthip et al., 2012; Best, 2017; Grant et al., 2016), we nevertheless decided to proceed with the validation of these negative regulators. qRT-PCR analysis indeed showed that the negative regulators of JAK/STAT signaling, *E(bx)* and *Socs36E*, were significantly upregulated in ZIKV-infected *Drosophila* flies at both 4 dpi (Fig. 3A) and 8 dpi (Fig. 3B). RNA-seq data were also validated through qRT-PCR of immunity-related genes, and, in particular, showed that *Tep1*, *TotM* and *Diedel* were strongly upregulated, whereas *CecA1* and *Dipt* were downregulated (Fig. 3C,D). Thus, qRT-PCR analysis showed a good correlation with RNA-seq data and suggests that *Drosophila* can be a suitable model for studying host-ZIKV interaction, where ZIKV can trigger unique processes including negative regulation of the well-characterized JAK/STAT signaling pathway.

### ZIKV transgene expression triggers retarded eye growth linked with reduced levels of JAK/STAT signaling

The demonstration of upregulation of *Et*, *E(bx)* and *Socs36E* through qRT-PCR analysis prompted us to probe the functional significance of the altered expression of these genes, particularly in the context of ZIKV transgene-induced eye development. In order to examine the effect of ZIKV on *Drosophila* eye development, we took advantage of genetic overexpression constructs. Gal4/UAS-based studies in *Drosophila* have led to identification of the functions of several viral genes, facilitating a comprehensive understanding of the outcome of viral infection (Hughes et al., 2012).

**Fig. 4.**
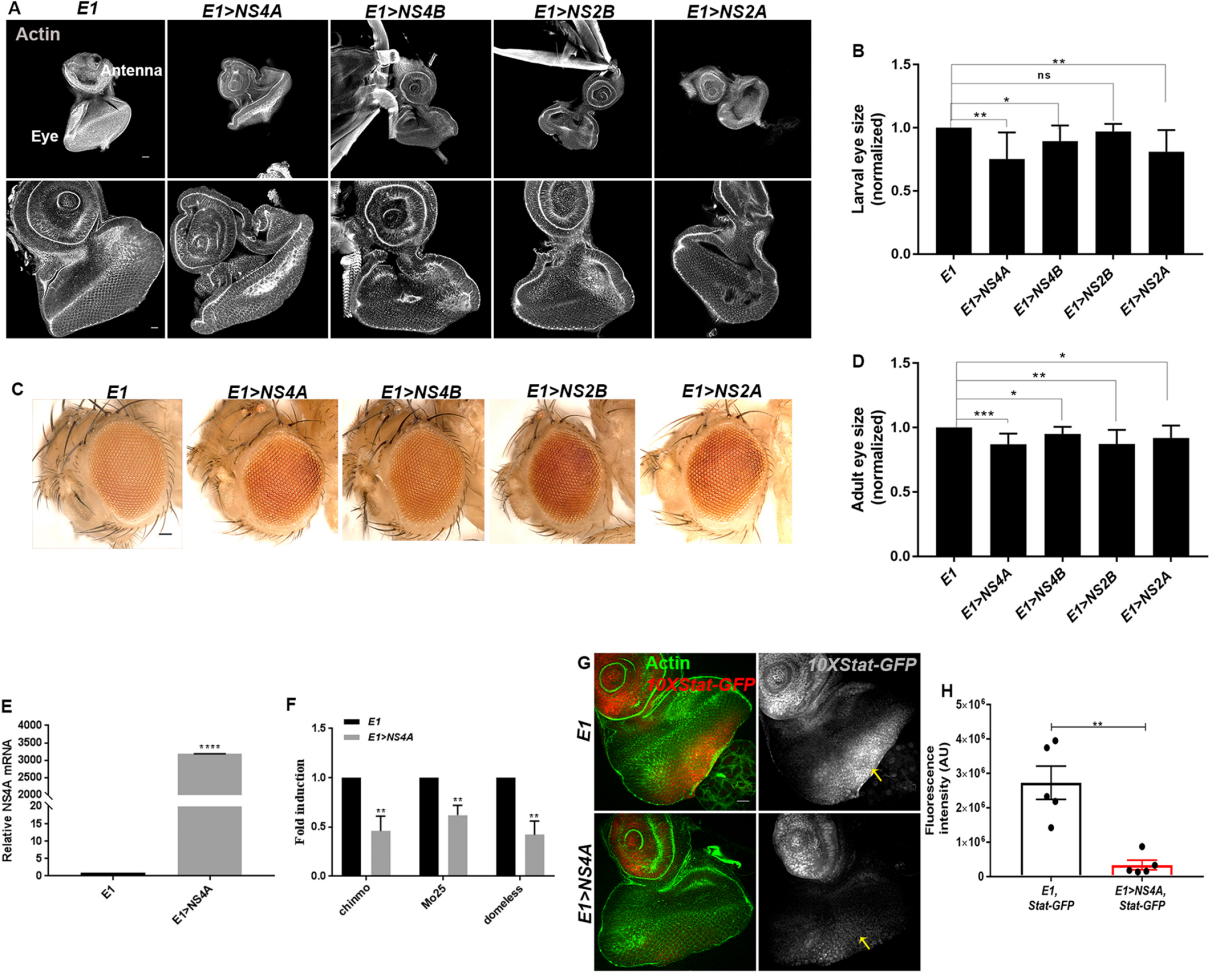
**ZIKV non-structural proteins induce restricted eye growth and are linked with downregulated JAK/STAT signaling.** (A) Representative eye imaginal discs upon overexpression of ZIKV non-structural protein-coding genes driven under eye-specific *eyeless-Gal4* (referred to as *E1-Gal4*) (*E1>NS4A*, *E1>NS4B*, *E1>NS2B* and *E1>NS2A*)*.* Lower row shows the enlarged view of the eye imaginal discs. The cytoarchitecture was marked with Actin (gray). (B) Quantification of the size of eye imaginal discs upon *E1-Gal4* driven overexpression of ZIKV non-structural protein coding genes, compared to *E1-Gal4* alone*.* Bars show mean±s.d. (*P<0.05, **P<0.01; ns, not significant). (C) Representative images of the adult eye in the indicated genotypes. (D) Quantification of the size of adult eye upon overexpression of ZIKV non-structural protein-coding genes, compared to *E1-Gal4*. Bars show mean±s.d. (***P=0.0001, **P=0.0016, *P<0.05). (E) qRT-PCR analysis depicting the mRNA level of *NS4A* in the eye imaginal epithelia overexpressing ZIKV *NS4A* driven under *E1-Gal4* (*E1>NS4A*), compared to *E1-Gal4* alone. Bars show mean±s.d. (****P<0.0001). (F) qRT-PCR analysis depicting the mRNA level of *chinmo*, *Mo25* and *domeless* in the eye imaginal disc where ZIKV *NS4A* was overexpressed. All data were normalized to the housekeeping gene *RpL32* and are shown relative to *E1-Gal4*. Three independent experiments were carried out in triplicate and bars represent mean±s.d. (**P<0.05). (G) Expression of *10XStat92E-GFP* (abbreviated to *10XStat-GFP*, shown by red in merge and gray in separate channel) in eye imaginal epithelia carrying *eyeless*-specific overexpression of ZIKV NS4A compared to *E1-Gal4* alone. The cytoarchitecture is marked with Actin (green). The *10XStat-GFP* levels are marked by yellow arrows in both *E1-Gal4*- and *E1>NS4A*-carrying eye imaginal epithelia. (H) Quantification of *10XStat-GFP* fluorescence intensity in the eye imaginal disc proper from *E1-Gal4*- and *E1>NS4A*-carrying larvae. Bars show mean±s.d. (**P=0.0015). Statistical analysis was performed using unpaired two-tailed Student's *t*-test. AU, arbitrary units. Scale bars: 100 μm.

Along with the viral genes that encode structural proteins, the non-structural proteins are also critical because they interact with the host cells to promote viral pathogenesis. Non-structural proteins of flaviviruses play crucial roles in their replication (Bollati et al., 2010). ZIKV, like other flaviviruses, possesses seven non-structural proteins: NS1, NS2A, NS2B, NS3, NS4A, NS4B and NS5 (Shi and Gao, 2017; Hasan et al., 2018). Here, we would like to emphasize that although the perturbation of JAK/STAT signaling was revealed from the infection dynamics of the whole fly, we switched to the *Gal4/UAS*-based genetic system to validate this finding and further explore the functions of ZIKV NS proteins. We generated genetic constructs overexpressing four of these non-structural proteins, namely *UAS-NS2A*, *UAS-NS2B*, *UAS-NS4A* and *UAS-NS4B.* To score for the developmental defect upon ZIKV NS protein overexpression, we crossed these constructs with an eye-specific, *eyeless-Gal4* (referred to as *E1-Gal4*), where Eyeless is one of the most critical transcription factors regulating eye development in *Drosophila* (Pallavi et al., 2012). Overexpression of genes encoding ZIKV *NS2A*, *NS2B*, *NS4A* and *NS4B* under *E1-Gal4* did not result in any survival defects (Fig. S1A). Scoring for the time of pupariation further showed that these flies did not exhibit any developmental delay (Fig. S1B). However, compared to *E1-Gal4*, overexpression of genes encoding ZIKV non-structural proteins *NS2A*, *NS2B*, *NS4A* and *NS4B* driven under *E1-Gal4* resulted in significantly reduced size of the eye imaginal disc, a phenotype also resulting from loss of function of *hop* (Perrimon and Mahowald, 1986; Mukherjee et al., 2005) (Fig. 4A,B).

We further examined whether ZIKV-induced infection affects retinal differentiation and found that eye imaginal epithelia from *E1-Gal4*-driven overexpression of *NS2A*, *NS2B*, *NS4A* and *NS4B* did not display any defect in the expression of pan-neural marker, Elav, which marks the retinal neuron-specific fate (Fig. S2). In corroboration with the reduced size of eye imaginal epithelia, we noticed that the resulting adult flies harboring *E1-Gal4*-mediated ZIKV non-structural protein overexpression also displayed a significant reduction in eye size (Fig. 4C,D). We noticed that ZIKV-induced reduction in adult eye size was very similar to the ‘small eye’ phenotype triggered upon loss of function of *unpaired* (*upd*) (Tsai and Sun, 2004; Bach et al., 2003). To ascertain that the eye phenotype is indeed a consequence of ZIKV transgene expression, we examined the transcriptional status of *NS4A* in the eye imaginal epithelia carrying *E1-Gal4*-mediated *NS4A* overexpression. Assigning the arbitrary value of 1 to the level of *NS4A* in the control eye imaginal epithelia (*E1-Gal4*), there was 3000-fold enrichment of *NS4A* in the eye imaginal epithelia when NS4A was overexpressed (Fig. 4E).

Given the similarity between ZIKV- and JAK/STAT-induced eye phenotypes, we next aimed to confirm whether the ZIKV-induced eye phenotype is indeed linked to impaired JAK/STAT signaling. qRT-PCR analysis revealed that the eye imaginal epithelia carrying *E1-Gal4*-driven *NS4A* overexpression showed significantly reduced expression of the targets of JAK/STAT signaling – including *chinmo*, *Mo25* and *domeless* – compared to *E1-Gal4* (Flaherty et al., 2009) (Fig. 4F). For a more robust validation, we switched to the *in vivo* reporter, *10XStat92E-GFP*, which accurately reflects the activation of the JAK/STAT pathway in several tissues (Bach et al., 2007). *10XStat92E-GFP* is expressed throughout the development of *Drosophila*, including the embryo and larval stages, in a pattern overlapping with the expression pattern of Stat92E protein (Bach et al., 2007). Moreover, it is activated by ectopic JAK/STAT signaling and there is loss of reporter expression in *Stat92E* clones (Bach et al., 2007). The control eye imaginal disc showed *10XStat92E-GFP* expression restricted to the antenna and the posterior compartment of the eye imaginal epithelia (Fig. 4G). Interestingly, overexpression of eye-specific ZIKV NS4A protein resulted in a substantial reduction of *10XStat92E-GFP* expression (Fig. 4G). We also noticed that this reduction was autonomous, as NS4A overexpression had no effect on the expression pattern of *10XStat92E-GFP* in the antenna of the eye imaginal disc. We further quantified the fluorescent intensity of the STAT reporter in the *E1*- and *E1>NS4A*-carrying eye imaginal epithelia and found a significant loss of STAT reporter activity where ZIKV NS4A was overexpressed (Fig. 4H). These findings suggest that ZIKV-regulated eye growth in *Drosophila* is linked with the substantial downregulation of the JAK/STAT signaling pathway.

### ZIKV-induced eye phenotype is associated with a reduced rate of proliferation without affecting the rate of apoptosis

**Fig. 5.**
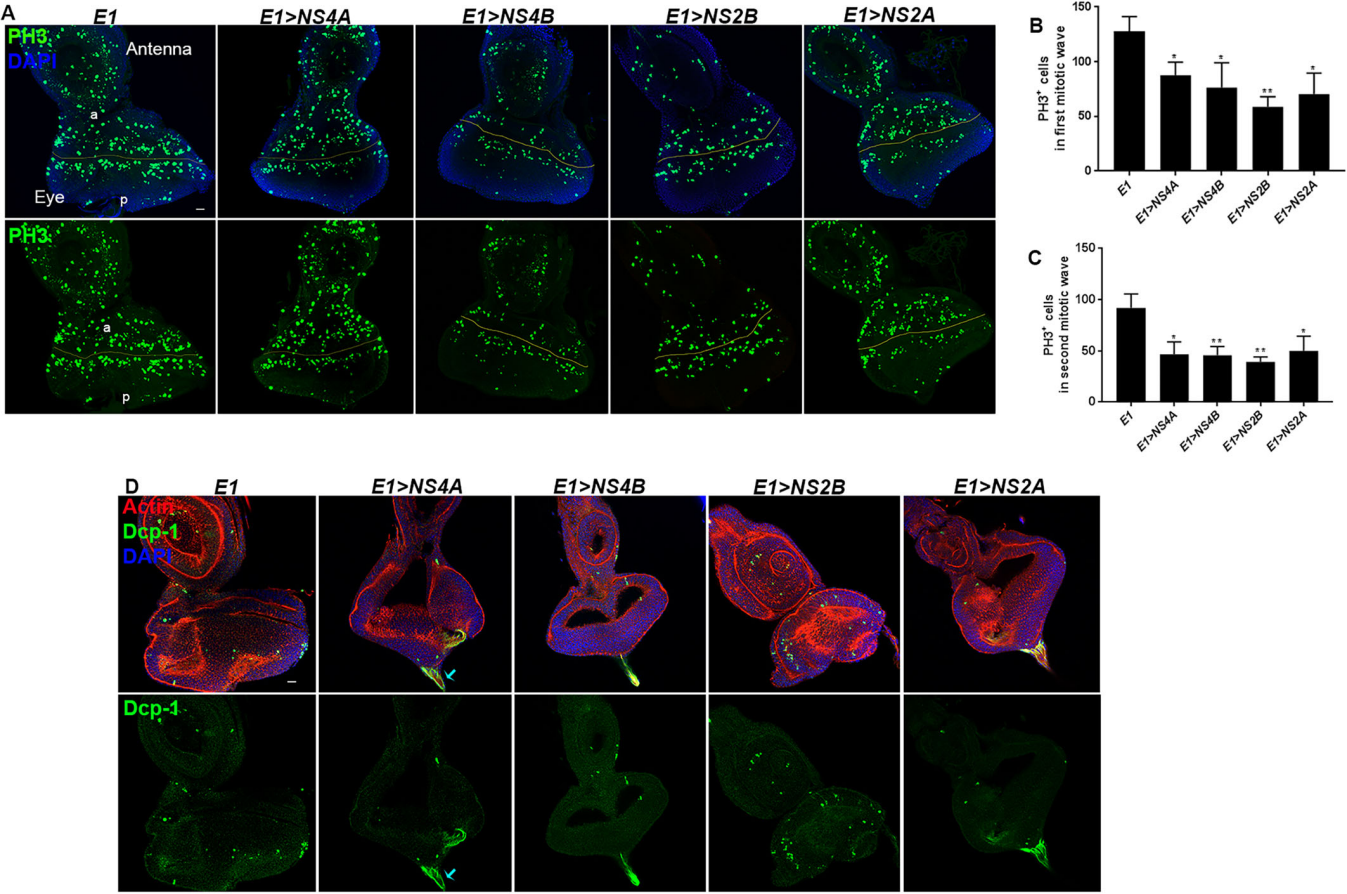
**ZIKV non-structural protein overexpression results in reduced rate of proliferation in eye imaginal epithelia.** (A,D) Representative eye imaginal discs overexpressing ZIKV non-structural protein-coding genes driven under eye-specific *E1-Gal4* (*E1>NS4A*, *E1>NS4B*, *E1>NS2B* and *E1>NS2A*). (A) The rate of proliferation was marked with anti-phosphohistone (PH3) staining (green). The yellow lines mark the morphogenetic furrow. The compartments are marked: a, anterior; p, posterior. In all images, nuclei were stained with DAPI (blue). (B) Quantification of the number of PH3-marked cells in the first mitotic wave or anterior region of eye discs upon ZIKV non-structural protein overexpression compared to *E1-Gal4* alone. Bars show mean±s.d. (*P<0.05, **P<0.01). (C) Quantification of the number of PH3-marked cells in the second mitotic wave or posterior region of eye discs upon ZIKV non-structural protein overexpression compared to *E1-Gal4* alone. Bars show mean±s.d. (*P<0.05, **P<0.01). (D) Cell death was indicated by anti-Dcp-1 staining (green); Actin was used to mark the cytoarchitecture (red). In all images, nuclei were stained with DAPI (blue). Statistical analysis for the graphs were performed using unpaired two-tailed Student's *t*-test. Scale bars: 100 μm.

We next examined the eye imaginal epithelia overexpressing ZIKV non-structural proteins for the overall rate of proliferation. We used the pan mitosis-marker, anti-phosphohistone (PH3), for detecting the mitotically active cells. We noticed numerous PH3-positive cells in both the anterior (first mitotic wave) and posterior compartment (second mitotic wave) in the eye imaginal epithelia in the case of *E1-Gal4* (Fig. 5A). Upon overexpression of eye-specific ZIKV non-structural proteins, the number of PH3-positive cells was reduced significantly (Fig. 5A). Quantification analysis showed that cells from both the first (Fig. 5B) and second (Fig. 5C) mitotic wave responded in a similar manner, and the total number of PH3-positive cells in the eye imaginal epithelia overexpressing ZIKV non-structural proteins was reduced to 50% of the PH3-positive cells in *E1-Gal4* alone (Fig. 5B,C).

We next examined the rate of apoptosis as one of the cellular factors contributing to the reduced size of the eye imaginal epithelia overexpressing ZIKV non-structural proteins. Unlike proliferation, we did not notice any reduction in the level of apoptosis in the eye imaginal epithelia overexpressing ZIKV non-structural proteins compared to *E1-Gal4* alone (Fig. 5D). Interestingly, however, ZIKV non-structural protein overexpression did trigger enhanced cell death in the optic stalk as shown by increased accumulation of anti-Dcp-1 (Fig. 5D). Together, these findings suggest that, at the cellular level, ZIKV infection triggers reduction in overall proliferation rate in the eye imaginal epithelia without significantly affecting apoptosis.

### Knockdown of *Stat* along with NS4A overexpression leads to synergistic reduction in eye size, while NS4A overexpression rescues Hop-mediated eye overgrowth

**Fig. 6.**
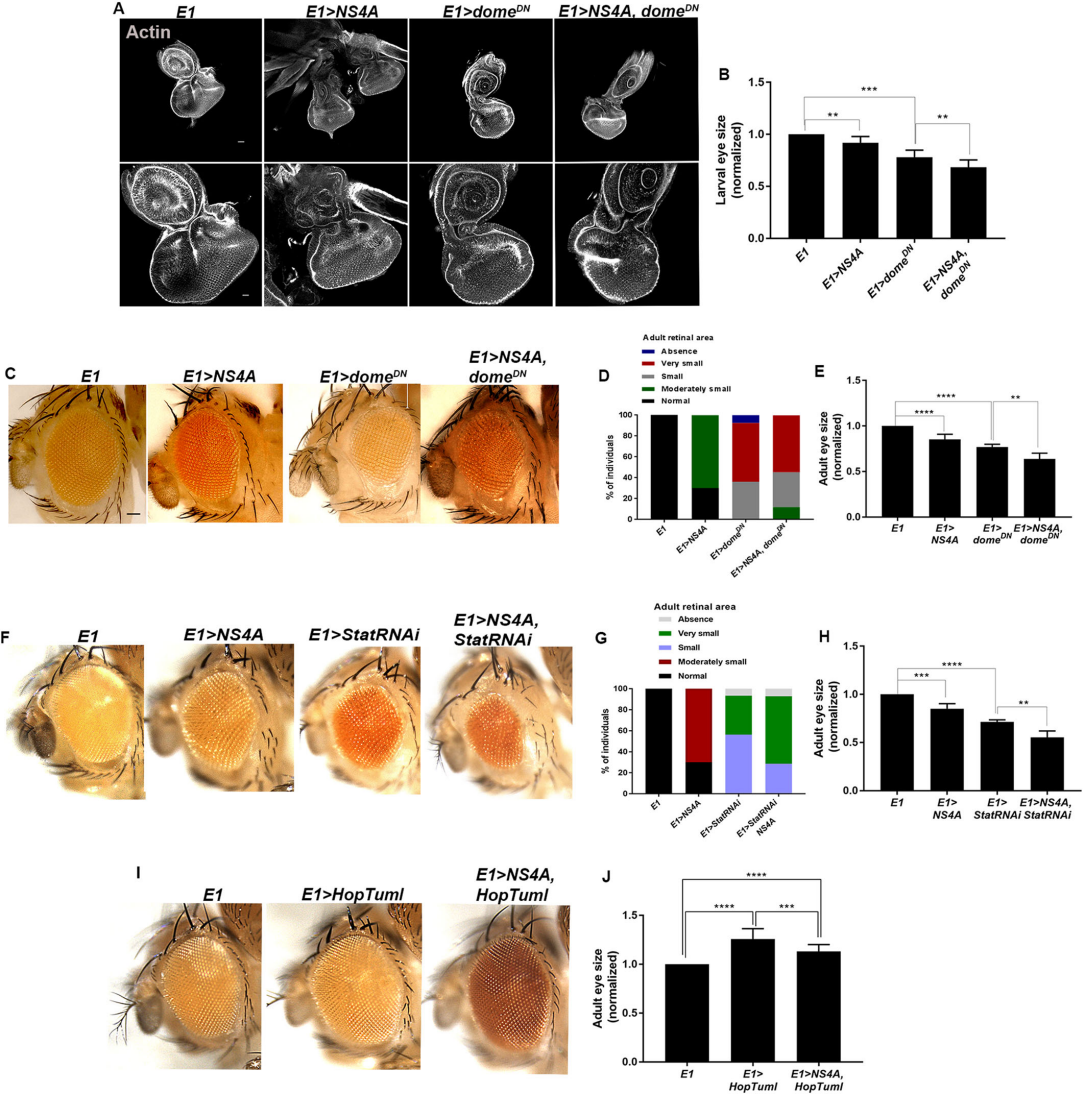
**ZIKV NS4A shows genetic interaction with different components of JAK/STAT signaling pathway.** (A) Representative eye imaginal discs overexpressing ZIKV *NS4A*, a dominant negative form of *domeless*, and co-expression of the dominant-negative form of *domeless* and *NS4A* driven under the eye-specific driver, *E1-Gal4* (*E1>NS4A*,* E1>dome^DN^* and *E1>NS4A*,* dome^DN^*, respectively), compared to *E1-Gal4* alone. The lower row shows the enlarged view of the eye imaginal discs. Cytoarchitecture was marked with Actin (gray). (B) Quantification of the size of eye imaginal discs in the indicated genotypes. Bars show mean±s.d. (***P<0.0001, **P<0.05). (C) Representative images of the adult eye in *E1>NS4A*,* E1>dome^DN^* and *E1>NS4A*,* dome^DN^*, respectively. (D) Percentage of individuals displaying normal, moderate, small, very small or absence of adult retinal area in the indicated genotypes. (E) Quantification of the size of adult eye in *E1>NS4A*,* E1>dome^DN^* and *E1>NS4A*,* dome^DN^*, respectively, compared to *E1-Gal4* alone. Bars show mean±s.d. (****P<0.0001, **P=0.0053). (F) Representative images of the adult eye overexpressing ZIKV *NS4A*, *StatRNAi* and co-expression of *StatRNAi* and *NS4A* driven under eye-specific *E1-Gal4* (*E1>NS4A*,* E1>StatRNAi* and *E1>NS4A*,* StatRNAi*, respectively). (G) Penetrance of different eye phenotypes in the indicated genotypes. (H) Quantification of the size of adult eye in *E1>NS4A*,* E1>StatRNAi* and *E1>NS4A*,* StatRNAi*, respectively, compared to *E1-Gal4* alone. Bars show mean±s.d. (****P<0.0001, ***P<0.001 and **P=0.0071). (I) Representative images of the adult eye upon *E1-Gal4*-driven overexpression of *HopTuml* and co-expression of *NS4A* and *HopTuml* (*E1>HopTuml* and *E1>NS4A*,* HopTuml*, respectively). (J) Quantification of the size of adult eye in *E1>HopTuml* and *E1>NS4A*,* HopTuml*, compared to *E1-Gal4* alone. Bars show mean±s.d. (****P<0.0001 and ***P=0.0006). Statistical analysis was performed using unpaired two-tailed Student's *t*-test. Scale bars: 100 μm.

After validating the status of JAK/STAT signaling in the developing eye tissue carrying overexpression of ZIKV NS4A protein, we next investigated the genetic interaction of ZIKV NS4A with the regulators of JAK/STAT signaling. We took advantage of estimating the adult as well as larval eye size as the assay for scoring genetic interaction. We genetically recombined the overexpression construct of ZIKV non-structural gene *NS4A* (*UAS-NS4A*) and dominant-negative form of Upd receptor, Domeless (*UAS-dome^DN^*) (Brown et al., 2001). The overexpression of the dominant-negative form of *domeless* driven under *E1-Gal4* (*E1>dome^DN^*) resulted in a drastic reduction in the size of the eye imaginal epithelia (Tsai and Sun, 2004) (Fig. 6A). Introduction of the dominant-negative form of *domeless* (*UAS-dome^DN^*) in the background of *E1-Gal4*-driven *NS4A* (*E1>NS4A*) overexpression resulted in aggravated reduction in eye size (Fig. 6A,B).

The adult fly carrying the overexpression of dominant-negative form of *domeless* under *E1-Gal4* displayed variable phenotype, ranging from complete absence of the eye to small and very small eye size (Fig. 6D). Compared to *NS4A* overexpression alone, where most of the flies showed moderately small eye size (Fig. 6C,D), introduction of the dominant-negative form of *domeless* in the background of *E1-Gal4*-mediated *NS4A* overexpression resulted in a range of eye phenotypes (Fig. 6C,D). The majority of the flies showed either small or very small eye size, while the flies bearing moderately sized eye reduced to 12% of the total number of flies tested (Fig. 6D). Quantification analysis further validated that the co-expression of *UAS-dome^DN^* and ZIKV *NS4A* overexpression aggravates the reduction in eye size compared to expression of *E1>UAS-NS4A* or *E1>UAS-dome^DN^* alone (Fig. 6E). We further validated this synergistic reduction by co-expressing *StatRNAi* and ZIKV NS4A overexpression using eye-specific *E1-Gal4*. Similar to *domeDN*, *E1>StatRNAi* resulted in reduced size of the adult eye and also demonstrated penetrance of a range of adult eye phenotypes ranging from very small to total absence of eye (Fig. 6F,G). Consistent with the role of Stat in eye development, clonal loss of *Stat92E* results in a small or ablated eye (Ekas et al., 2006). Quantification of the size of adult eye showed that *eyeless*-specific overexpression of ZIKV *NS4A* and *StatRNAi* resulted in significantly smaller eyes than *E1>NS4A* or *E1>StatRNAi* alone (Fig. 6H).

To further validate the genetic interaction between ZIKV NS4A and JAK/STAT signaling, we then overexpressed JAK/STAT signaling components and asked if co-expression of ZIKV NS4A could rescue the STAT-mediated phenotype. Ectopic misexpression of the JAK/STAT ligand, Upd, using *E1-Gal4* (*E1>upd*) results in enlarged eye phenotype (Bach et al., 2003) (Fig. S3). However, after co-expression of ZIKV NS4A and *upd* together in the developing eye, we still observed an enlarged eye (Fig. S3). We then switched to a less-severe eye phenotype to examine the effect of ZIKV NS4A overexpression. Overexpression of activated Hop kinase, *HopTuml* (also known as *hop^Tum^*), also results in enlargement of the eye (Bach et al., 2003) (Fig. 6I,J). When we co-expressed ZIKV *NS4A* and *HopTuml* under *E1-Gal4*, we noticed that there was a significant, although modest, reduction in the size of the developing eye (Fig. 6I,J). Thus, the aggravation of the *StatRNAi*- and *domeDN*-induced eye phenotype and the partial rescue of the *HopTuml*-mediated overgrowth, confirms the genetic interaction between JAK/STAT signaling components and ZIKV *NS4A.*

### ZIKV transgene expression fails to regulate the growth of posterior compartment of larval wing epithelia but *engrailed*-specific NS4A overexpression triggers thickening of veins in adult wing

Apart from regulating eye development, JAK/STAT signaling also regulates wing development and patterning. The mutation in *upd* resulting in ‘*outstretched*’ phenotype is characterized by the outstretched wing posture in the adult fly. The outstretched wings phenotype was also associated with the upregulation of a negative regulator of JAK/STAT signaling, Socs36E (Callus and Mathey-Prevot, 2002). Driven under a wing-specific Gal4, directed expression of Socs36E further resulted in loss of the wing anterior cross vein, formation of wing vein deltas and humeral outgrowths (Callus and Mathey-Prevot, 2002). In a recent study, JAK/STAT was also shown to regulate the posterior compartment of wing imaginal epithelia and promote cycling and survival of these cells (Recasens-Alvarez et al., 2017). The JAK/STAT-mediated reduced size of the posterior compartment was further attributed to the decreased expression of cell cycle marker, CycA, and increased apoptosis (Recasens-Alvarez et al., 2017).

**Fig. 7.**
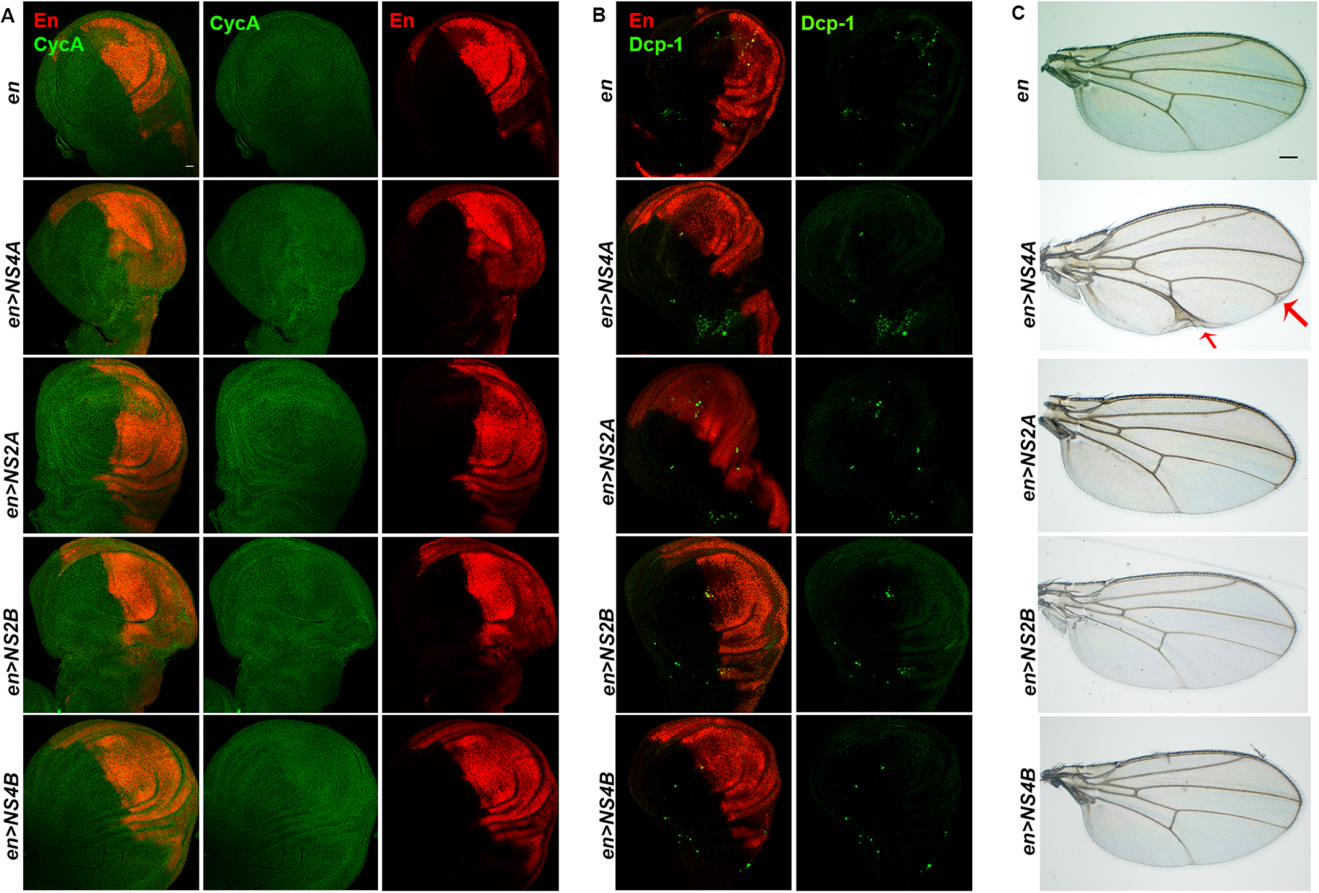
**ZIKV NS4A overexpression results in thickening of veins in adult wing.** (A) Representative wing imaginal discs upon overexpression of ZIKV non-structural protein-coding genes driven under wing-specific, *engrailed-Gal4* (*en>NS4A*, *en>NS2A*, *en>NS2B* and *en>NS4B*)*.* The engrailed marked compartment was tagged with RFP (*enGal4>UAS-RFP*). Wing disc-specific JAK/STAT target, CycA, is marked in green. (B) Anti-Dcp-1 was used to show cell death (green) in the indicated genotypes. (C) Representative images of the adult wing in *en>NS4A*, *en>NS2A*, *en>NS2B* and *en>NS4B*. The thickening and branching of veins are depicted with red arrows. Scale bars: 100 μm.

We next investigated if ZIKV transgene expression triggers wing defects in *Drosophila*. Based on JAK/STAT signaling-induced wing phenotypes, ZIKV non-structural proteins were overexpressed using a wing-specific *engrailed-Gal4* [Engrailed (En) marks the posterior compartment of the wing]. We noticed that there was no difference in the size of the posterior compartment (tagged with RFP), compared to the anterior compartment of the wing imaginal disc (shown by the absence of RFP), when *engrailed*-*Gal4*-specific ZIKV non-structural proteins were expressed (Fig. 7A). We also did not encounter any noticeable change in the level of CycA in the two compartments, compared to the anterior compartment of wing imaginal disc, when *engrailed-Gal4*-specific ZIKV non-structural proteins were expressed (Fig. 7A). We further examined apoptotic activity in the posterior compartment and found that ZIKV non-structural protein overexpression failed to trigger any change in the level of apoptosis (Fig. 7B). These findings in the larval stage were further consistent in the adult stage, when the eclosed flies did not show any defect in the size of the adult wing (Fig. 7C). Interestingly, overexpression of ZIKV NS4A, however, resulted in a very reproducible and highly penetrant (90% of the flies, *n*=50) phenotype marked by thickening of veins and formation of wing deltas (Fig. 7C), a phenotype characteristic of Socs36E overexpression (Callus and Mathey-Prevot, 2002). These findings suggest that while ZIKV non-structural proteins unambiguously regulate eye growth, the wing phenotype is mainly restricted to the overexpression of ZIKV NS4A.

### ZIKV NS4A regulates wing growth linked with downregulated Notch signaling

**Fig. 8.**
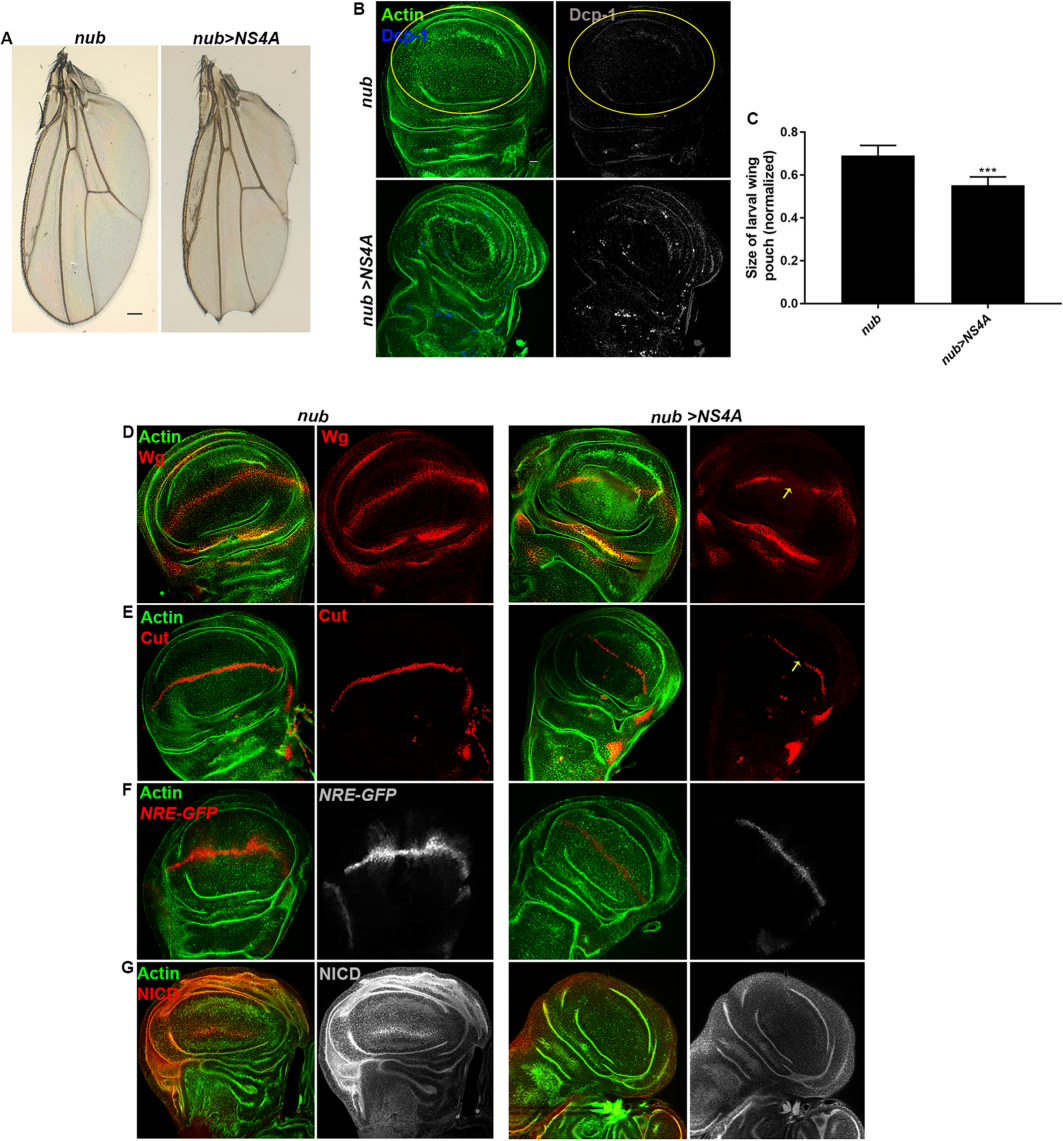
**ZIKV NS4A overexpression results in notching of wing linked with downregulated Notch signaling.** (A) Representative images of the adult wing upon overexpression of ZIKV NS4A driven under wing-specific, *nubbin-Gal4* (*nub>NS4A*), compared to *nub-Gal4* alone. (B-E) Representative wing imaginal discs displaying overexpression of ZIKV NS4A driven under *nub-Gal4* (*nub>NS4A*), compared to *nub-Gal4* alone. For clarity, the nubbin region is marked with a yellow outline. (B) Cell death was indicated with anti-Dcp-1 staining (blue in merge panel and shown in gray in separate channel). (C) Quantification of the size of the wing pouch upon overexpression of ZIKV non-structural protein NS4A under *nub-Gal4* (*nub>NS4A*), compared to *nub-Gal4* alone*.* The size of the wing pouch in each genotype was normalized with respect to the size of the whole wing disc. Bars show mean±s.d. (***P=0.0001). (D,E) Wingless and Cut, the targets of Notch signaling, are marked in red. Reduced expression of Wg and Cut upon NS4A overexpression (*nub>NS4A*) is indicated by yellow arrows. (F) Overexpression of NS4A in the wing pouch (*nub>NS4A*) significantly downregulated Notch signaling reporter *NRE-GFP* expression at the dorsal/ventral boundary, compared to *nubbin-Gal4* alone. *NRE-GFP* is shown in red in the merge images and in gray in the separate channel. (G) The amount of Notch intracellular domain (NICD) was significantly reduced when ZIKV NS4A was overexpressed using *nubbin-Gal4* (*nub>NS4A*), compared to *nubbin-Gal4* alone. Notch protein level was marked with anti-NICD staining (red in merge channel and gray in the separate channel). In all images, cytoarchitecture was marked with Actin (green). Scale bars: 100 μm.

We noticed that ZIKV NS4A-induced wing vein thickening is also a characteristic phenotype of mutation in Notch signaling. This prompted us to examine other possible wing defects upon overexpression of ZIKV non-structural proteins. We used *nubbin-Gal4*, where Nubbin is specific to the pouch domain of the wing imaginal disc, to specify the proximo-distal axis in *Drosophila* wing (Ng et al., 1995; Neumann and Cohen, 1998; Cifuentes and Garcia-Bellido, 1997). Nubbin-driven overexpression of NS4A resulted in a classic notched wing phenotype marked by loss of wing margin (Fig. 8A). Furthermore, this wing phenotype was highly reproducible and penetrant (85% of the flies, *n*=55). Similar to the *engrailed-Gal4* findings, we found that overexpression of *NS2A*, *NS2B* and *NS4B* had no noticeable defect in the adult wing (Table S6). The classical notched wing phenotype is attributed to downregulated Notch signaling. We next examined the underlying mechanism for NS4A-induced notched phenotype. We found that *nub-Gal4*-driven *NS4A* expression resulted in drastic reduction in the size of the pouch region of the wing imaginal epithelia (Fig. 8B,C). In addition, the wing imaginal epithelia displayed increased apoptosis as marked by increased expression of Dcp-1 (Fig. 8B).

We then investigated the status of Notch signaling and found that ZIKV NS4A overexpression (*nub>NS4A*) substantially reduces Wg and Cut expression in the pouch domain of the wing imaginal epithelia, where *wg* and *cut* are the morphogens and are considered the targets of Notch signaling (Neumann and Cohen, 1996; Giraldez and Cohen, 2003) (Fig. 8D,E). We further validated the status of Notch signaling using the reporter *NRE-GFP*, which consists of a Notch responsive element (NRE) fused with a GFP reporter (Saj et al., 2010). The expression of GFP is restricted to the dorsal/ventral (DV) boundary and its expression thus reliably marks the cells with active Notch signaling. Corroborating with reduced expression of Wg and Cut, we found that *NRE-GFP* was significantly reduced when ZIKV NS4A was overexpressed in the *nubbin* region (marks the wing pouch encompassing the DV boundary) of the wing imaginal epithelia (Fig. 8F). To determine if Notch is reduced by overexpression of NS4A, we examined the expression level of Notch protein in wing disc carrying *nubbin*-specific overexpression of ZIKV NS4A. Antibody staining against the Notch receptor, Notch intracellular domain (NICD), showed that, similar to the expression pattern of Cut, Wg and *NRE-GFP*, there was reduced expression of Notch protein when ZIKV NS4A was overexpressed compared to *nubbin-Gal4* alone (Fig. 8G).

## DISCUSSION

Host-virus interaction and its effect on host physiology has so far remained mostly unexplored. Recently, studies based on transcriptomic and proteomic analyses have been instrumental in advancing our understanding of how viruses can affect host physiology (Merkling et al., 2015; Sun et al., 2017; Dong et al., 2017). Our results further emphasize the applicability of such transcriptomic analyses in understanding host-virus interactions and their molecular regulators. Here, we have shown that infection of adult *Drosophila* with the MR766 strain of ZIKV results in differential expression of genes implicated in crucial biological processes. Furthermore, analyzing the RNA-seq by putting a stringent filter cutoff resulted in just a handful of biological processes, whereas the number of biological processes increased considerably only when a modest filter cutoff was employed.

Our results show that ZIKV infection induces some of the generic stress-mediated responses, including enrichment of genes related to response to oxidative stress, upregulation of the Turandot family of genes and induction of *Diedel* (Harsh et al., 2018). However, ZIKV infection does not trigger the activity of heat shock proteins, unlike DCV infection (Merkling et al., 2015).

Infertility is considered another infection-induced pathology where in order to sustain the fitness of an individual, the reproducing ability is reduced (Shirasu-Hiza and Schneider, 2007). Infection with Flock house virus causes oocyte destruction, marked by degenerating egg chambers, disorganized posterior follicle cells and reduction in fecundity (Thomson et al., 2012). Our RNA-seq analysis shows that the genes associated with vitelline membrane and eggshell assembly – such as *Cp36*, *nudel*, *Femcoat*, *dec-1* and *Cp7Fc* – are downregulated upon ZIKV infection, while others have reported that mutation in *Cp36* and *dec-1* results in female sterility (Hawley and Waring, 1988; Mauzy-Melitz and Waring, 2003).

In mammals, interferon-mediated JAK/STAT signaling is considered to be antiviral in nature. Various strategies of IFN-induced JAK/STAT antagonism have been shown for flaviviruses. Dengue virus infection results in loss of STAT2 expression, while West Nile virus infection results in failed JAK activation in an attempt to evade JAK/STAT-mediated immunity (Guo et al., 2005; Jones et al., 2005). *In vitro* studies indicate that, similar to dengue virus, ZIKV NS5 binds to STAT2 and targets it for degradation (Grant et al., 2016). Accumulating evidence indicates that the contribution of JAK/STAT signaling is virus specific in *in vivo* models like *Drosophila* and mosquitos. Flies mutant for JAK are more sensitive to cricket paralysis virus (CrPV) and DCV; however, these mutant flies show a rather weak phenotype to *Drosophila* X virus (DXV), Sindbis virus (SINV) and vesicular stomatitis virus (VSV) (Kemp et al., 2013; Dostert et al., 2005). Whereas activation of JAK/STAT restricts dengue virus infection, it does not impart resistance in *Aedes aegypti* to ZIKV or Chikungunya virus infection (Jupatanakul et al., 2017; Souza-Neto et al., 2009).

In the case of ZIKV infection in *Drosophila*, a recent finding indicates that one of the negative regulators of JAK/STAT signaling, *Diedel*, is significantly enriched in ZIKV-infected flies (Harsh et al., 2018). The upregulation of Diedel throughout the stages of infection could be a potential strategy of ZIKV to evade JAK/STAT-mediated immune response. Our findings showing the upregulation of negative regulators Eye transformer (Et) and Socs36E, in addition to reduced level of JAK/STAT reporter, *10XStat92E-GFP*, highlight the complex nature of this interaction and indicate that ZIKV infection in *Drosophila* also inhibits the development-induced JAK/STAT signaling. *Et* is structurally related to *Drosophila* JAK/STAT receptor, *Domeless*, and *Et* knockdown triggers hyperactivation of septic injury-induced JAK/STAT targets and enhances unpaired-induced eye overgrowth (Kallio et al., 2010). The Suppressor of cytokine signaling (Socs) genes are one of the best-characterized JAK/STAT pathway negative regulators and constitute one of the first feedback loops identified in JAK/STAT signaling (Kile and Alexander, 2001). Overexpression of Socs36E, in particular, phenocopies the outstretched phenotype of *upd* mutants and the venation defects associated with *Stat* allele (Rawlings et al., 2004).

The intracellular nature of infection and the complexities linked with host-virus interaction has made it difficult to decipher the pathways responsible for symptoms or disease. *Drosophila* and its strong genetic tools enable a spatial- and temporal-based genetic manipulation and therefore have emerged as an excellent model system to dissect host-virus interactions (Hughes et al., 2012). Expression of a viral transgene in a tissue-specific manner has led to the deciphering of the functions of several viral components in terms of interaction with host signaling pathways or factors (Hughes et al., 2012). Flavivirus non-structural proteins are considered to be the most critical for their replication and assembly. The host and viral protease-mediated cleavage of flaviviral polyprotein results in seven non-structural proteins, which share structural and functional similarity among the different members of the family (Apte-Sengupta et al., 2014; Bollati et al., 2010). fNSCs expressing ZIKV NS4A and NS4B display impaired neurogenesis and elevated autophagy, which in turn has been linked with inhibited Akt-mTOR signaling (Liang et al., 2016). In line with these findings, our results show that eye-specific expression of ZIKV non-structural proteins, specifically NS4A, results in markedly restricted size of the developing eye. The consistency of the eye phenotype throughout the larval and the adult stage strongly indicates that ZIKV non-structural proteins interact with the regulatory proteins involved in *Drosophila* eye development. Indeed, our findings validating the reduced level of JAK/STAT signaling and genetic interaction between ZIKV NS4A and JAK/STAT signaling components further imply that ZIKV pathogenesis is linked with eye development, which in turn is associated with regulation of JAK/STAT signaling. These observations also become more relevant in the light of evidence that, unlike other flaviviruses, ZIKV can also be found in the eyes, reproductive organs and bodily secretions, including saliva and urine (Gourinat et al., 2015; Musso et al., 2015; Sun et al., 2016).

As intracellular pathogens, viruses devise strategies to ensure their own proliferation and persistence at the cost of host growth. Some viruses facilitate cell proliferation while others are known to inhibit cell proliferation and in turn result in infection-induced pathologies. Human T cell leukemia virus and human papilloma viruses, for example, encode proteins that promote cell proliferation and result in oncogenic transformation (Op De Beeck and Caillet-Fauquet, 1997). HIV viral protein R (Vpr) is one of the best-characterized proteins known to inhibit T-cell clonal expansion and support the replication of the virus (Poon et al., 1998; Zhao and Elder, 2005; Zhao et al., 2011). Emerging evidence indicates that ZIKV can infect neural precursor cells and subsequently inhibit proliferation and induce apoptosis (Wang et al., 2018; Li et al., 2016a,b). The effects of ZIKV on cell proliferation have also been suggested to be the underlying mechanism for ZIKV-induced pathologies, including microcephaly. Our data depicting the impaired proliferation in the developing eye overexpressing ZIKV transgene further stress the interaction between ZIKV and host cell proliferation. The reduced rate of proliferation could also be the cellular basis for the ZIKV-induced restricted growth of the eye. Future investigations will focus on how reduced cell proliferation benefit ZIKV replication and result in adverse effects to the host.

In the context of neuropathology and ZIKV infection, a recent study demonstrated that overexpression of ZIKV NS4A results in reduced size of larval brain in *Drosophila* (Shah et al., 2018). It was further shown that ZIKV-induced microcephaly is mediated through human Ankryin repeat and LEM domain-containing 2 (ANKLE2). Interestingly, *Drosophila* eye epithelia are attached to the optic lobes of the brain. During larval development, while the morphogenetic furrow is developing in the eye, the receptor neurons are specified (Freeman, 1997; Silies et al., 2007). These neurons project their axons to the posterior end of the eye disc, resulting in the optic stalk, which in turn connects the eye disc with optic lobes of the brain. Mutation in the gene *disco*, responsible for proper formation of the optic stalk, results in defective innervation of neurons from the eye disc to the brain (Steller et al., 1987; Holmes et al., 1998). Given the close proximity of the eye and brain lobes, and our observation that there is an increased accumulation of cell death in the optic stalk (connects the brain and developing eye) upon overexpression of ZIKV NS4A, further suggests that ZIKV transgene expression in *Drosophila* can also result in neurological abnormalities. Future investigations could provide insight into the neurological pathologies of ZIKV transgene expression.

Our results further show that, in addition to the eye, ZIKV transgene expression can also affect *Drosophila* wing and regulate its development and patterning. Our findings indicate that ZIKV infection-induced growth regulation is maintained in *Drosophila* wing as well. However, unlike the eye, the outcome of ZIKV infection is compartment specific in wing. While ZIKV NS4A overexpression does not affect the growth of the posterior compartment of the wing, ZIKV NS4A expression in the pouch domain (encompassing the dorsal/ventral compartment) results in the restricted growth of this domain. The subdivision of the wing into anterior/posterior (a/p) compartments occurs in the embryonic stage, while the DV boundary is established during proliferation of the imaginal disc (Garcia-Bellido et al., 1973). ZIKV effect on the pouch domain without affecting the posterior compartment thus emphasizes the regulation of cell proliferation by ZIKV-induced infection. In *Drosophila* wing, Notch signaling is crucial in regulating cell differentiation and patterning. Notch signaling, in particular, regulates expression of several genes involved in the DV boundary and elicits wing cell proliferation (Irvine and Vogt, 1997). Our finding that ZIKV transgene-mediated effect on wing growth is regulated via reduced Notch signaling is suggestive of the role of ZIKV-induced infection in regulating cell proliferation in the wing imaginal epithelia as well.

Taken altogether, our results illustrate the efficacy of *Drosophila* as a model for understanding the pathology resulting from host-ZIKV interactions. Our findings reveal that ZIKV plays a crucial role in regulating the growth of *Drosophila* wing and eye and results in their reduced size. Whereas ZIKV-induced pathogenesis in the eye is linked with reduced JAK/STAT signaling, in the wing it correlates with reduced level of Notch signaling. The key findings presented in this study will assist in unraveling ZIKV-induced pathogenesis and will advance our understanding of host-ZIKV interactions.

## MATERIALS AND METHODS

### Fly stocks

The following fly lines were used: *w^1118^* (wild-type control), *E1-Gal4* (Pallavi et al., 2012), *engrailed Gal4UAS-RFP* (Bloomington no. 30557), *UAS-NS4A*, *UAS-NS4B*, *UAS-NS2A*, *UAS-NS2B*, *UAS-StatRNAi* (Vienna *Drosophila* Resource Center no. 43866), *w^1118^; P(10XStat92E-GFP)* (Bloomington no. 26197), *w^1118^; P(NRE-EGFP.S)5A* (Bloomington no. 30727), *nubbin-Gal4* (Bloomington no. 25754). *UAS-upd* and *UAS-HopTuml* were generously provided by Heinrich Jasper (Buck Institute for Research and Aging, Novato, CA, USA), and *UAS-dome^DN^* was provided by Dan Hultmark (Umeå University, Umeå, Sweden). Flies were reared on instant *Drosophila* diet (Formula 4-24 *Drosophila* medium) supplemented with yeast (Carolina Biological Supply), and maintained at 25°C and in a 12:12-h light:dark photoperiodic cycle. Female adult flies aged 4-6 days old were used in infection assays with ZIKV.

### DNA cloning and generation of transgenic fly strains

Four ZIKV open reading frames (ORFs) (NS2A, NS2B, NS4A and NS4B) in mammalian expression vectors were gifts from Dr Hongjun Liu's laboratory in Johns Hopkins University (Yoon et al., 2017). To generate Flag-tagged UAS-ZIKV-ORF constructs, each ZIKV ORF was PCR amplified with specific primers using mammalian expression vector as template. Each amplicon was inserted into the pUASTattB vector with EcoRI and KpnI restriction enzymes and introduced into the germ cells of flies by standard P element**-**mediated germ line transformation. Primers used for PCR were as follows: ZIKV-NS2A: forward, 5′-CCG***GAATTC***ATGGATTACAAGGATGACGACGATAAGGGGTCAACCGATCATATGGAC-3′; reverse, 5′-CGG***GGTACC***CTACCGCTTCCCACTCCTTGTGAG-3′. ZIKV-NS2B: forward, 5′-CCG***GAATTC***ATGGATTACAAGGATGACGACGATAAGAGCTGGCCCCCTAGTGAAGTTC-3′; reverse, 5′-CGG***GGTACC***CTACCTTTTCCCAGTCTTCACATAC-3′. ZIKV-NS4A: forward, prime;-CCG***GAATTC***ATGGATTACAAGGATGACGACGATAAGGGAGCGGCTTTGGGAGTAATG-3′; reverse, 5′-CGG***GGTACC***CTATCTTTGCTTCTCTGGCTCGGG-3′. ZIKV-NS4B: forward, 5′-CCG***GAATTC***ATGGATTACAAGGATGACGACGATAAGAACGAACTTGGATGGCTGGAAAG-3′; reverse, 5′-CGG***GGTACC***CTAACGTCTCTTAACCAGGCCAGC-3′. Bold italic sequences are restriction enzyme cutting sites and underlined sequences are Flag tag.

### ZIKV infection

The ZIKV infection protocol and propagation have been described in detail previously. Briefly, the African strain of ZIKV, MR766, was propagated in Vero cells followed by determination of ZIKV titers using plaque assay on Vero cells as described in Delvecchio et al. (2016). For fly infection, adult female flies were intrathoracically injected with 100 nl live ZIKV solution [11,000 plaque-forming units (PFU)/fly] using a nanoinjector (Nanoject III, Drummond Scientific). Injection of the same volume of PBS served as a negative control. Injected flies were kept at 25°C and transferred to fresh vials every third day. They were collected at the different timepoints and directly processed for RNA analysis.

### RNA isolation

Total RNA was extracted from ten adult female flies injected with ZIKV and PBS at 4 and 8 dpi in triplicate using Trizol according to the manufacturer's protocol. Total RNA was re-suspended in 30 μl sterile nuclease-free water. RNA concentration was measured using Nanodrop. RNA integrity and quality were assessed on formaldehyde agarose gel.

### Library preparation and RNA-seq

TruSeq Total RNA with Ribo-Zero Gold Kit (Illumina, San Diego, CA, USA) was used to generate strand-specific RNA-seq libraries following the manufacturer's recommendations. The libraries were pooled and sequenced in a single lane on an Illumina HiSeq 2500 sequencing instrument with single-end 50 bp reads. RNA-seq was performed at The Genome Technology Access Center (GTAC) based in Washington University in St Louis, MO, USA.

RNA-seq reads were aligned to the Ensembl release 76 top-level assembly with STAR version 2.0.4b (Dobin et al., 2013). Gene counts were derived from the number of uniquely aligned unambiguous reads by Subread: feature Count version 1.4.5 (Liao et al., 2014). Transcript counts were produced by Sailfish version 0.6.3 (Patro et al., 2014). Sequencing performance was assessed for total number of aligned reads, total number of uniquely aligned reads, genes and transcripts detected, ribosomal fraction known junction saturation and read distribution over known gene models with RSeQC version 2.3 (Wang et al., 2012). All RNA-seq data have been deposited in NCBI Gene Expression Omnibus (GEO) with the accession number GSE130108.

### Bioinformatics analysis

All gene counts were then imported into the R/Bioconductor package EdgeR (Robinson et al., 2010) and TMM normalization size factors were calculated to adjust for samples for differences in library size across samples after ribosomal genes. Genes not expressed greater than 1 count per million in at least two samples were removed from further analysis. The TMM size factors and the matrix of counts were then imported into R/Bioconductor package Limma and weighted likelihoods based on the observed mean-variance relationship of every gene/transcript and sample were calculated for all samples with the voomWithQualityWeights function (Liu et al., 2015). Generalized linear models were then created to test for gene/transcript level differential expression. DEGs and transcripts were then filtered for FDR adjusted *P*-values less than or equal to 0.1.

### GO analysis

GO analysis was performed using DAVID 6.8 bioinformatics resources (http://david.abcc.ncifcrf.gov/). In all cases, analyses were performed using the list of DEGs. The *P*-value cutoff to determine enriched pathways was 0.1.

### qRT-PCR validation

To validate DEGs, we selected eight candidate genes based on significant fold differences and analyzed their mRNA levels using qRT-PCR. Ten adult female flies injected with PBS or ZIKV were frozen at 4 and 8 days dpi. Total RNA was extracted using Trizol according to the manufacturer's protocol. Total RNA (350-500 ng) was used to synthesize cDNA using a High-Capacity cDNA Reverse Transcription Kit (Applied Biosystems). qRT-PCR experiments were performed in technical triplicates and with gene-specific primers (Table 1) using iQ SYBR Green Supermix (Bio-Rad) and a CFX96 Real-Time PCR detection system (Bio-Rad). Quantification was performed from three biological replicates for both test and control treatments. Fold changes were calculated with the delta-delta Ct method using *RpL32* as a housekeeping gene.

**Table 1.**
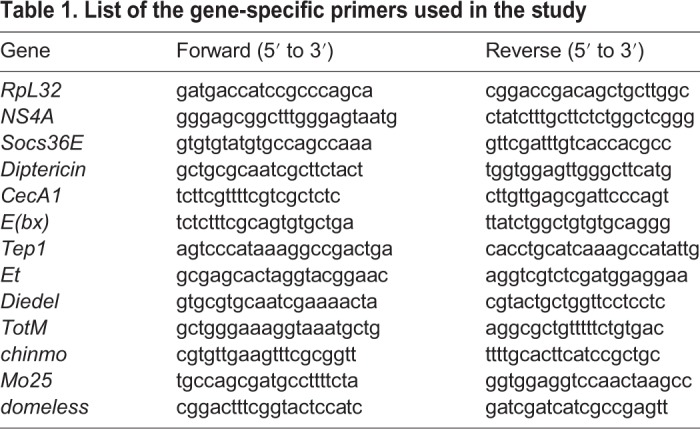
**List of the gene-specific primers used in the study**

### Fly survival

For each fly strain, three groups of 20 female flies carrying *eyeless*-specific overexpression of non-structural proteins were used. Flies were maintained at a constant temperature of 25°C with a 12-h light/dark cycle, and mortality was recorded daily. Log-rank (Mantel–Cox) test was used to analyze the survival curves.

### Pupariation delay

Two-hour timed egg collections were carried out and L1 larvae were collected 24 h after egg deposition and reared at 30 animals per vial. The number of larvae that had pupariated at a given time after egg deposition was scored every 8 h.

### Immunostaining and antibodies

Anti-PH3 (1:500; Abcam), anti-CycA [1:50; Developmental Studies Hybridoma Bank (DSHB)], anti-Dcp-1 (1:100; Cell Signaling Technology), anti-Cut (1:10; DSHB), anti-Wg (1:1000; DSHB), anti-Elav (1:50; DSHB) and anti-NICD (1:10; DSHB) were used as the source for primary antibodies. Secondary antibodies included Alexa Fluor 488, 555 and 633 (Invitrogen). For nuclear staining, 4′,6-diamidino-2-phenylindole (DAPI; Invitrogen) was used. Standard procedures were followed for immunostaining. The crosses were properly synchronized for the stages and third-instar larvae were collected for dissections. Briefly, fly tissues were dissected and fixed in PBS containing 4% formaldehyde for 30 min. Following double rinsing in PBS containing 0.1% Triton X-100, the samples were incubated overnight at 4°C with the primary antibody. The samples were then blocked with 1% BSA for 2 h followed by a 2-h incubation with secondary antibody at room temperature. Finally, the samples were mounted with Vectashield medium (Vector Laboratories). Images were acquired with a Zeiss LSM 510 confocal microscope and processed using Adobe Photoshop CS6.

### Fluorescence quantification

Relative amounts of fluorescence were measured with ImageJ software by using Shanbhag thresholding on images and calculating the integrated density, resulting area and mean fluorescence of the background. The following equation was used: corrected total fluorescence=integrated density−(area×mean fluorescence of background).

### Larval wing imaginal disc and adult wing size

The size of the third-instar wing imaginal discs and adult wings were measured using ImageJ. In the case of the wing pouch, the size in different genotypes was normalized to the total size of the wing disc.

### Adult eye imaging

The photographs and measurements were acquired using a Keyence VHX 5000 Digital Microscope.

### Statistical analysis

qRT-PCR results represent the means±s.d. of relative values from three biological replicates. An unpaired two-tailed Student's *t*-test was used for statistical analysis of data using Prism (GraphPad Software).

## Supplementary Material

Supplementary information
