# Supplementary material for: Zika virus non-structural protein NS4A restricts eye growth in Drosophila through regulation of JAK/STAT signaling
Source: Dis Model Mech. 2020 Apr 30;13(4):dmm040816. doi: 10.1242/dmm.040816 (PMC7197722; doi:10.1242/dmm.040816)
Supplement: Supplementary information [file dmm-13-040816-s1.pdf]

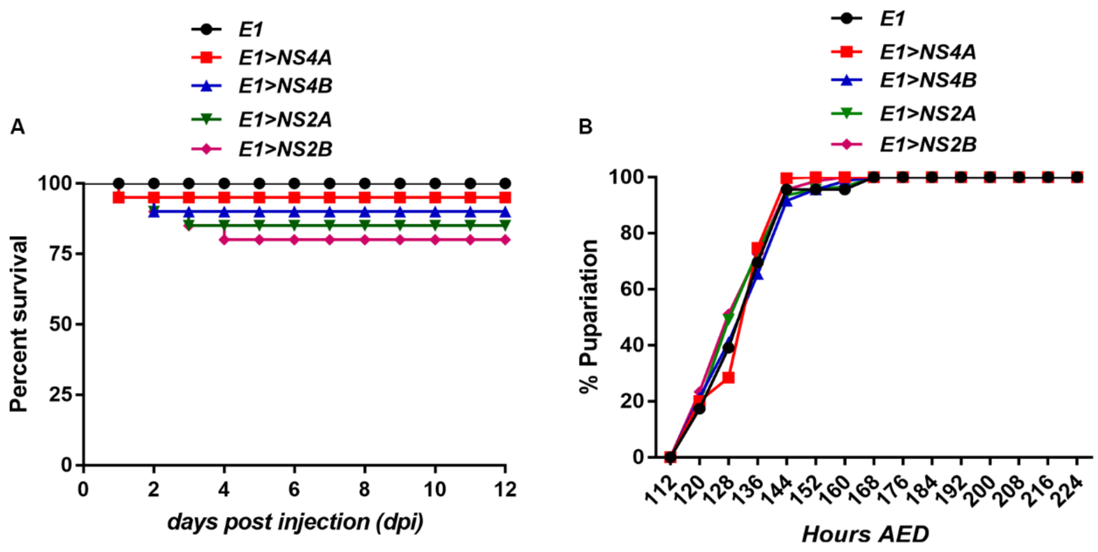

Fig. S1. ***Eyeless*** **specific overexpression of ZIKV non-structural (NS) proteins confers no overall effect on host development.** (A) Survival of adult flies carrying *eyeless* specific overexpression of ZIKV NS proteins (*E1> NS4A, E1>NS4B, E1>NS2A* and *E1>NS2B*) as compared to *E1-Gal4* alone. The flies were monitored for a duration of 12 days post eclosion at 24-hr intervals. All the colored lines depicting the survival of the denoted genotype are superimposed, but for clarity, they are shown in parallel. Log-rank (Mantel–Cox) was used for statistical analysis. (B) *Eyeless* specific overexpression of ZIKV NS proteins has no effect on the pupariation times. Percentage of larvae that have pupariated at the indicated time point is shown (n = 30, triplicate experiments).

Disease Models & Mechanisms • Supplementary information

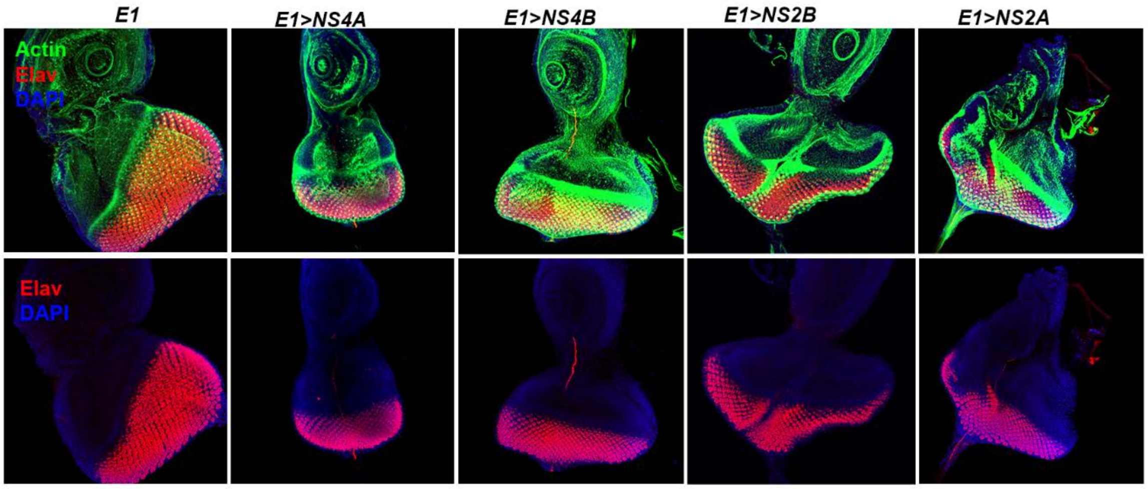

Fig. S2. Overexpression of ZIKV non-structural proteins does not affect the differentiation of developing eye. Representative eye imaginal discs upon overexpression of ZIKV non-structural protein coding genes (*UAS-NS4A*, *UAS-NS4B*, *UAS-NS2B* and *UAS-NS2A*) driven under eye-specific *eyeless-Gal4 (E1-Gal4), (E1>NS4A, E1>NS4B, E1>NS2B* and *E1>NS2A).* In all images, cytoarchitecture is marked with actin (green), photoreceptor with Elav (red) and nuclei with DAPI (blue).

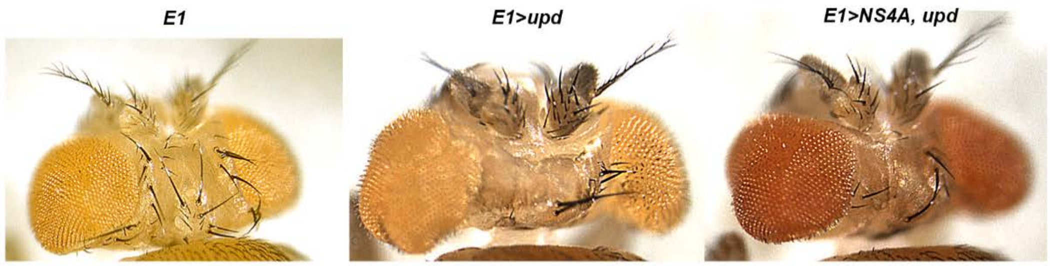

Fig. S3. *Eyeless* specific overexpression of NS4A is not sufficient to rescue the Upd induced enlargement of the eye. Representative images of the adult eye overexpressing *upd* (*E1>upd*) and co-expression of *NS4A* and *upd* (*E1>NS4A*, *upd*) driven under eye-specific *eyeless-Gal4* as compared to *E1-Gal4* alone.

Disease Models & Mechanisms • Supplementary information

Table S1: Upregulated genes triggered upon ZIKV infection in adult *Drosophila* at 4dpi.

List of the upregulated genes with the cut off log2 fold change ≥ 1.2 and adjusted p<0.1**.**

Click here to Download Table S1

Table S2: Downregulated genes triggered upon ZIKV infection in adult *Drosophila* at 4dpi.

List of the downregulated genes with the cut off log2 fold change ≤ -1.2 and adjusted p<0.1.

Click here to Download Table S2

Table S3: Upregulated genes triggered upon ZIKV infection in adult *Drosophila* at 8dpi.

List of the upregulated genes with the cut off log2 fold change ≥ 1.2 and adjusted p<0.1.

Click here to Download Table S3

Table S4: Downregulated genes triggered upon ZIKV infection in adult *Drosophila* at 8dpi.

List of the downregulated genes with the cut off log2 fold change ≤ -1.2 and adjusted p<0.1.

Click here to Download Table S4

Table S5: Log FC and adjusted p value of *SOCS36E*, *E(bx)* and *Et* in the three biological replicates (adult flies injected with ZIKV or PBS at 4 and 8 dpi respectively).

4dpi	**Genes**	**log FC**	**adj p value**	**ZIKV 3**	**ZIKV 2**	**ZIKV 1**	**Sterile 3**	**Sterile 2**	**Sterile 1**
	E(bx)	1.987181	0.011	4.907868	3.699929	5.490515	-5.888	-4.57994	-4.695
	Et	3.310639	0.29	1.330568	2.639	2.984754	-4.26042	-0.14806	-1.65351
	SOCS36E	0.369518	0.76	5.441869	5.109804	5.510852	4.344573	4.667023	5.186825

8dpi	**Genes**	**log FC**	**adj p value**	**ZIKV 3**	**ZIKV 2**	**ZIKV 1**	**Sterile 3**	**Sterile 2**	**Sterile 1**
	E(bx)	4.796046	0.39	4.7643	3.9153	3.741208	-4.74671	4.824742	-4.78759
	Et	4.581048	0.73	2.53741	2.419358	0.432455	1.44494	0.317128	-4.78759
	SOCS36E	4.850252	0.88	-4.20262	3.204509	4.078984	-4.74671	2.342761	-4.78759

Table S6: Notching and size of adult wing in *nubbin-Gal4* driven overexpression of ZIKV NS2A (*nub>NS2A*), NS2B (*nub>NS2B*) and NS4B (*nub>NS4B*) proteins.

Genotype	Notching phenotype	Adult wing size/Wing size of *nub-Gal4* (%)
nub	No	100
nub>NS2A	No	102.34
nub>NS2B	No	104.76
nub>NS4B	No	104.12

Disease Models & Mechanisms • Supplementary information
